# The Contralateral Ankle Joint Is a Reliable Reference for Testing Syndesmotic Stability Using Bilateral External Torque CT

**DOI:** 10.1177/10711007241262771

**Published:** 2024-07-29

**Authors:** Anna-Katharina Calek, Esteban Ongini, Bettina Hochreiter, Reto Sutter, Stephan H. Wirth, Silvan Beeler

**Affiliations:** 1 Department of Orthopedics, Balgrist University Hospital, University of Zurich, Zurich, Switzerland; 2 Institute of Biomechanics, Balgrist Campus, ETH Zurich, Zurich, Switzerland; 3 Department of Radiology, Balgrist University Hospital, University of Zurich, Zurich, Switzerland

**Keywords:** syndesmosis, syndesmotic instability, external torque, ankle, ankle instability

## Abstract

**Background:**

Subtle chronic or latent instabilities are difficult to delineate with currently available diagnostic modalities and do not allow assessment of ligamentous functionality. The noninvasive bilateral external torque computed tomography (CT) was able to reliably detect syndesmotic lesions in a cadaveric study. The aim of the study was to test the external torque device in young, healthy subjects at 3 different torque levels and to demonstrate comparability with the contralateral side.

**Methods:**

Ten healthy subjects without history of injury or surgery to the ankle joint were enrolled in this cross-sectional study. Four CT scans were performed. During the scans, the lower legs and feet were placed in an external torque device with predefined external rotation torques of 0, 2.5, 5, and 7.5 Nm. Five different radiographic measures of syndesmotic stability were measured: anterior distance (AD), tibiofibular clear space (TCS), posterior distance (PD), external rotation (ER), and β angle.

**Results:**

With increasing external torque, slight increases in AD, ER, and β angle were observed, whereas TCS and PD decreased slightly. Large absolute differences were found between the healthy subjects for all measured parameters, regardless of the external torque applied. Differences from the contralateral side using the same external torque were minimal for all parameters, but smallest for AD with a maximum difference of 0.5 mm.

**Conclusion:**

Using the healthy contralateral ankle joint is appropriate for assessing syndesmotic stability based on minimal intraindividual side differences using the external torque device. Side differences >0.5 mm in AD and >0.9 mm in PD may be considered abnormal and may indicate significant instability of the syndesmosis. However, future studies are needed to define definitive cutoff values for relevant side differences in acute and chronic syndesmotic instability to guide clinicians in their treatment decisions.

## Introduction

Isolated distal tibiofibular syndesmotic injuries occur in approximately 1% to 17% of ankle sprains^7,9^ and up to 30% in high-impact sports.^10,23^ If left untreated, these injuries may lead to long-term disabilities such as chronic pain, instability, and early ankle osteoarthritis.^11,19,29^ However, assessment of the distal tibiofibular syndesmosis is difficult, especially in subtle chronic or latent instabilities.^2^ In these circumstances, conventional radiographs or fluoroscopy including stress views are inaccurate and do not reliably reflect the injury pattern, limiting their general applicability.^15,25^ Although magnetic resonance imaging (MRI) reveals ligament injuries and is therefore widely used in acute or chronic syndesmotic evaluation,^15^ it often provides inconclusive information about ligament functionality. One reason for this is the multiplanar nature of the problem, so that the instability can often only be diagnosed under load.^4,12-14^ Because of the large interindividual variability,^3,6,7,9^ several authors suggest comparing fibular translation with the contralateral side rather than using rigid cutoff values,^8,16,22,26^ and using external rotation rather than an axial force.^4,12,13,20,32^ Although several other studies have been applying external torque to the ankle joint to identify syndesmotic injuries,^28^ no standardized forces have been used, and definitive cutoff values have yet to be established.

Recently, Beeler et al^1^ presented an “external torque device” that gradually applies an external torque to both ankle joints during a CT scan. This “bilateral external torque CT” (BET-CT) overcomes many disadvantages of other diagnostic methods: it allows 3-dimensional (3D) assessment of the tibiofibular relationship and direct comparison with the contralateral side. With BET-CTs, syndesmotic lesions have successfully been diagnosed in a cadaver study.^1^

However, before the device is applicable in patients with syndesmotic injuries, comparability to the healthy contralateral side has to be proven by excluding relevant intraindividual differences. Therefore, the purpose of this study was to test the external torque device in young healthy subjects at 3 different torque levels. The hypothesis was that there would be no significant differences between the right and left ankle in healthy subjects.

## Methods

The study was approved by the responsible investigational review board. In this prospective study, we compared the left ankle joint with that of the right ankle joint using 4 different radiographic parameters. Ten healthy subjects without history of injury or surgery to the ankle joint were enrolled. Recruitment took place through an announcement in the institution where the study was conducted. The mean age was 27.2 years (range 24-32); 5 were males and 5 were females.

Subjects first underwent MRI (3T MAGNETOM Prisma, Siemens Healthineers, Erlangen, Germany) of both ankles to rule out preexisting ligamentous injuries of the anterior-inferior tibiofibular ligament (AITFL), posterior-inferior and transverse tibiofibular ligament (PITFL), superficial and deep deltoid ligament (DL) and fibular ligaments (anterior talofibular [ATFL], posterior talofibular [PTFL], and calcaneofibular [CFL] ligaments). The scans were interpreted by a board-certified musculoskeletal radiologist (R.S.).

**Figure 1.**
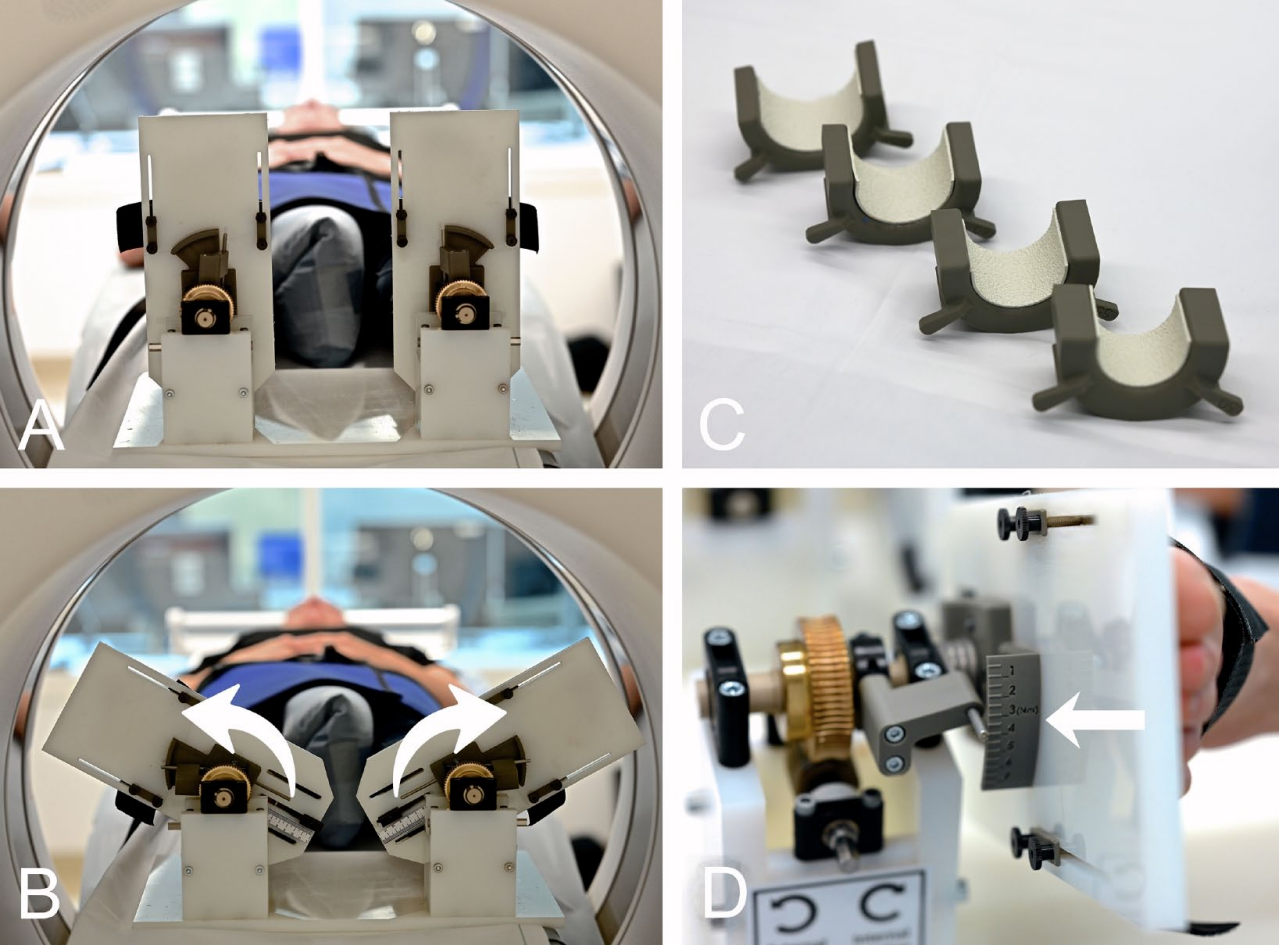
External torque device. (A) Ankle joints mounted in the external torque device in the neutral position without external torque. (B) Ankle joints mounted in the external torque device with external torque. (C) Interchangeable heel rests of different sizes. (D) Scale for reading the actual external torque applied.

**Figure 2.**
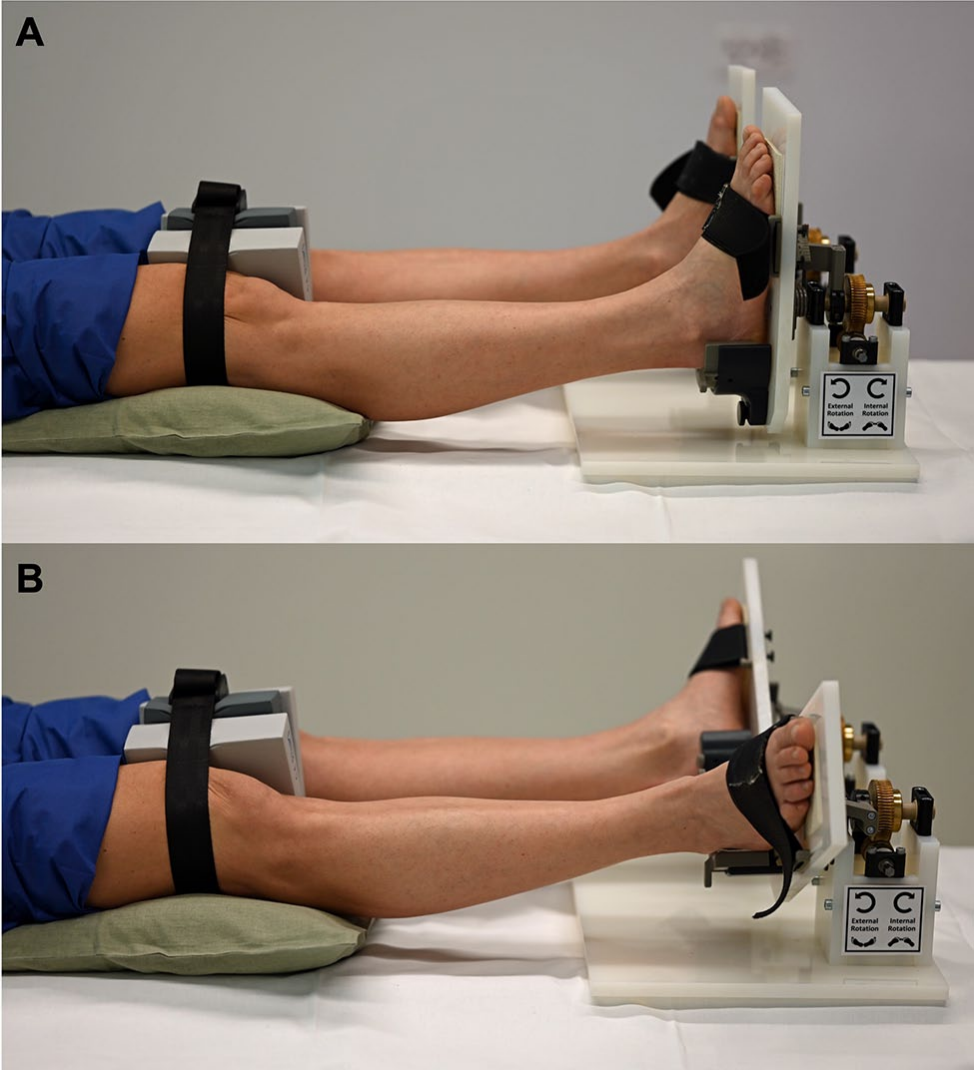
(A) Ankle joints mounted in the external torque device in the neutral position without external torque. A strap was used to reduce external rotation of the hip joints without contacting the proximal tibiofibular joint. (B) Ankle joints mounted in the external torque device with external torque.

Four CT scans (NAEOTOM Alpha, Siemens Healthineers, Erlangen, Germany) with a slice thickness of 0.8 mm were then performed. The lower legs and feet were placed in an external torque device (Figure 1A and B). Different sizes of heel rests were provided to create optimal test conditions, providing a comfortable neutral foot position without putting too much pressure on the heel itself, but also preventing the heels from moving too much during the test (Figure 1C). For testing, the lower legs were stabilized with a special knee brace to limit external rotation of the hip joints (Figure 2). A CT was performed in neutral ankle position (without torque), which served as a reference measurement, and then predefined external torques of 2.5, 5, and 7.5 Nm were applied via the external torque device. The healthy subjects were asked to relax; CTs were performed approximately 30 seconds after torque application.

The exclusion criteria for our study were defined as any signs of ankle instability, previous ankle or foot surgery or trauma on either side, ligamentous injuries detected by MRI, individuals under 18 years of age, and pregnancy. Inclusion criteria were defined as healthy subjects with no evidence or history of ankle instability, subjects older than 18 years and younger than 50 years, written informed consent.

### Bilateral External Torque CT

**Figure 3.**
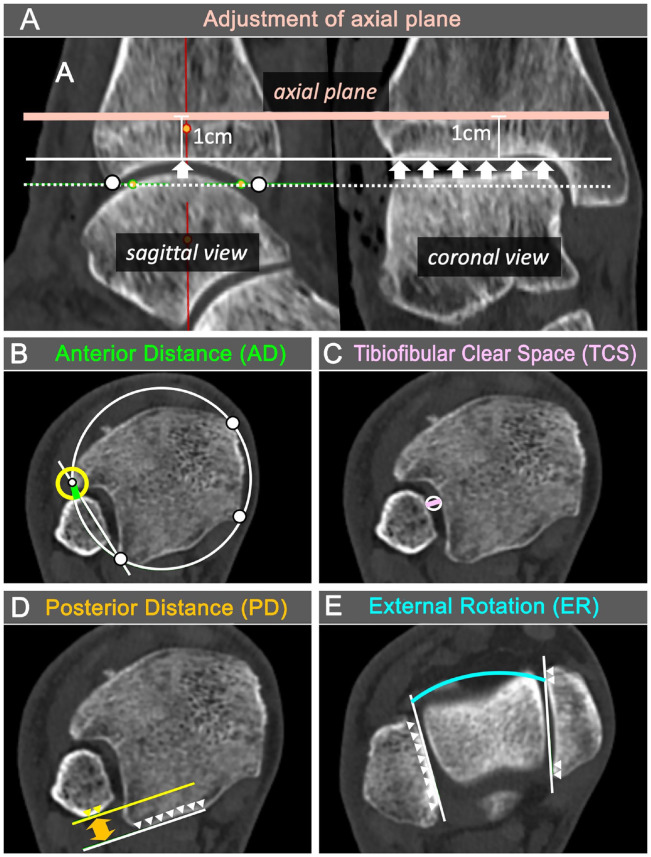
Computed tomography (CT) measurements to evaluate syndesmosis. (A) Sagittal and coronal CT images with plane used for measurements (salmon-colored solid line): 1 cm above the tibial plafond plane. It is parallel to the anterior-posterior pilon (white dotted line) and parallel to the coronal joint line (white arrows). (B) Anterior tibiofibular distance (AD) in green. Measured as the radius of a circle (yellow circle) centered between the best-fitting circle around the tibia (white circle, white touch points) and the tangent along the tibial notch (white solid line) just touching the fibula. (C) Tibiofibular clearance (TCS) in purple (purple solid line), measured as the diameter of the best-fitting circle (white circle) between the tibia and fibula at the center of the tibial notch. (D) Posterior tibiofibular distance (PD) in orange (orange arrow), measured between a tangent (white solid line) at the posterior margin of the tibia (white arrowheads) and a second line (yellow solid line) parallel to the first and just touching the posterior margin of the fibula (yellow arrows). (E) Fibular external rotation (ER) in turquoise, as the angle between the inner surface of the medial and lateral malleolus (white solid lines, white arrowheads). The β angle was measured similar to the ER measurement, but at the talar dome level. [See online article for color figure.]

The BET-CT procedure has been described in detail before^1^: CT scans were performed with external rotation forces of 0, 2.5, 5, and 7.5 Nm, objectified with a wrench. In addition, a scale on the device itself showed the torque being applied (Figure 1D). The Merlin PACS 5.2 Imaging software (Phoenix-PACS GmbH, Freiburg im Breisgau, Germany) was used for the calibration of 2 axial slices and the radiologic measurements. The axial alignment was first adjusted to the tibial plafond plane in the coronal and sagittal views (Figure 3A). An axial slice 1 cm proximal to the tibial plafond plane (proximal axial plane, Figure 3A) and a second axial plane distal to the tibial plafond plane (distal axial slice) were selected in the most proximal plane where the articular surface of the medial and lateral malleolus was just visible (Figure 3E).

Four different radiographic measures were measured in the 2 axial slices.^16-18,30^ The anterior tibiofibular distance (AD, in millimeters; Figure 3B) was measured in the proximal axial plane and defined as the distance between the intersection of a best-fitting circle around the tibia and a tangent along the tibial notch and the fibula. The tibiofibular clear space (TCS, in millimeters; Figure 3C) was measured in the proximal axial plane and defined as the distance at the center of the tibial notch. The posterior tibiofibular distance (PD, in millimeters; Figure 3D) was measured in the proximal axial slice and defined as the distance between a tangent at the posterior margin of the tibia and a second line parallel to the first just touching the posterior margin of the fibula. The fibular external rotation (ER, in degrees; Figure 3E) was measured in the distal axial slice and defined as the angle between the inner surface of the medial and lateral malleolus.

Based on the results of a previous study showing good overall agreement for CT measurements,^1^ interobserver agreement was not performed.

To assess the medial side, the β angle was measured according to Nault et al.^24^ The β angle is similar to the ER measurement (Figure 3E), but at the talar dome level.

## Statistical Analysis

An a priori power analysis was performed based on the cadaveric data to estimate the sample size required to demonstrate syndesmotic injuries with CT measurements. The smallest effect size observed in cadaveric experiments with at least a 2-ligament injury of the syndesmosis for AD was 1.20, corresponding to a mean difference of 1.02 mm in AD assuming the sample SD of 0.85 mm in the intact joint.^1^ For a Wilcoxon signed rank test with 80% power and 5% significance level, a minimum sample size of N = 8 would be required.

Visual data inspection of the data and Shapiro-Wilk tests revealed that some factor levels were nonnormally distributed. Consequently, nonparametric means were used for inference testing. Differences in CT measurements between the ipsilateral and contralateral leg were investigated using the Wilcoxon signed-rank test for each applied torque. The reliability of the contralateral measurement as the ipsilateral measurement was evaluated with intraclass correlation coefficients (ICCs), modeled with a 1-way random effects model with a single measurement per observation ICC (1, 1). Reference ranges for normal differences in CT measurements between ipsilateral and contralateral legs for healthy subjects were defined to lie within the 95% range of the data, values outside of the reference range were defined as abnormal. Data analysis was performed using GraphPad Prism 9 for Windows, version 9.2.0 (GraphPad Software LLC, San Diego, CA). Analysis of measurement reliability was performed using IBM SPSS Statistics for Windows, version 28.0 (IBM Corp, Armonk, NY). *P* <.05 was set for statistical significance

## Results

There were no significant differences in age between male and female participants (27.6 years for females vs 26.8 years for males; *P* = .604).

### CT Measurements Using the External Torque Device

**Figure 4.**
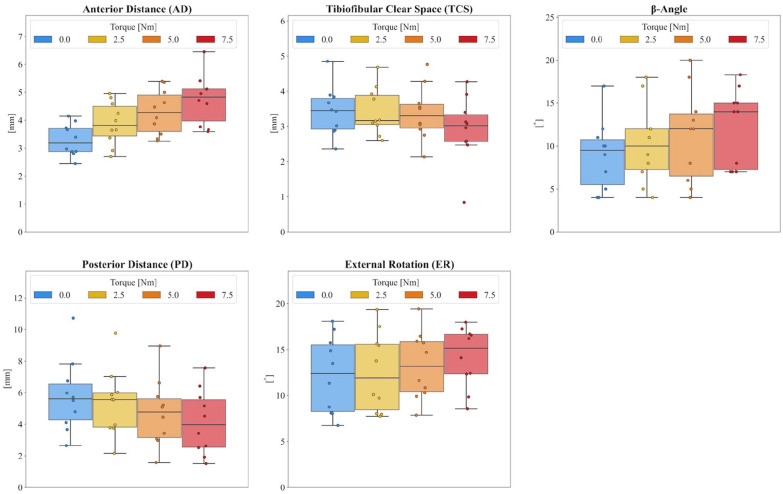
Computed tomographic measurements for all healthy subjects according to the amount of external torque (0-7.5 Nm).

**Figure 5.**
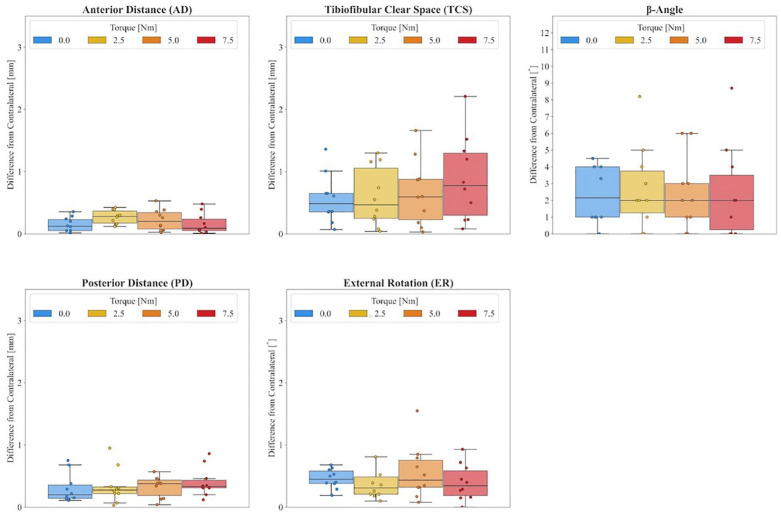
Absolute differences to the contralateral side according to the amount of external torque (0-7.5 Nm).

The medians and ranges of the CT measurements as a function of the external torque applied are shown in Table 1 and Figure 4. With increasing external torque, slight increases in AD, ER, and β angle were observed, whereas TCS and PD decreased slightly. The absolute differences to the contralateral side are shown in Table 2 and visualized in Figure 5. The 95% limits were calculated using all 3 external torques, which were ±0.48 mm for the AD, ±1.65 mm for the TCS, ±0.85 mm for the PD, ±1.52 degrees for the ER, and ±8.09 degrees for the β angle.

**Table 1.** Computed Tomographic Measurements According to the Amount of External Torque (0-7.5 Nm).

Torque	Anterior Distance, mm, Median (Range)	Tibiofibular Clear Space, mm, Median (Range)	Posterior Distance, mm, Median (Range)	External Rotation, degrees, Median (Range)	β Angle
0 Nm	3.2 (2.5–4.2)	3.5 (2.3–5.5)	5.6 (2.5–10.7)	12.2 (6.5–18.8)	7.9 (4–18)
2.5 Nm	3.8 (2.7–5.1)	3.3 (2.3–5.9)	5.5 (2.2–9.8)	12.1 (7.8–19.4)	8 (4–20)
5 Nm	4.1 (3.1–5.4)	3.3 (2.1–5.6)	4.8 (1.4–9.4)	12.9 (7.4–19.8)	10 (4–20)
7.5 Nm	4.8 (3.3–6.5)	3.1 (0.8–5.5)	4 (1.2–7.9)	15.2 (7.6–18.7)	10 (5–18.3)

**Table 2.** Absolute Differences to the Contralateral Side According to the Amount of External Torque (0-7.5 Nm) and Comparisons Between Left and Right Sides.^a^

Torque	Anterior Distance, mm, Median (Range)	*P* Value	Tibiofibular Clear Space, mm, Median (Range)	*P* Value	Posterior Distance, mm, Median (Range)	*P* Value	External Rotation, mm, Median (Range)	*P* Value	β Angle, Median (Range)	*P* Value
0 Nm	0.1 (0–0.4)	.922	0.5 (0.1–1.4)	.127	0.2 (0.1–0.8)	.492	0.5 (0.2–0.7)	.922	2.15 (0–4.5)	.266
2.5 Nm	0.3 (0.1–0.4)	.232	0.5 (0.0–1.3)	.084	0.3 (0.0–1.0)	.359	0.3 (0.1–3.1)	.900	2 (0–8.2)	.188
5 Nm	0.2 (0–0.5)	.242	0.6 (0.0–1.7)	.106	0.4 (0.0–0.6)	.287	0.4 (0.1–1.6)	.168	2 (0–6)	**.031**
7.5 Nm	0.1 (0–0.5)	.084	0.8 (0.1–2.2)	.065	0.3 (0.1–0.9)	.977	0.3 (0–0.9)	.999	2 (0–8.7)	.078

a Bold *P* values indicate statistical significance.

Differences from the contralateral side using the same external torque were minimal for all parameters, but smallest for AD with a maximum difference of 0.5 mm when higher torques were applied. Considering all torques, maximum differences of 2.2 mm, 1 mm, 3.1 degrees, and 8.7 degrees were observed for TCS, PD, ER, and β angle, respectively. There was no statistically significant difference in any of the paired left-right comparisons. Increasing the external torque had no effect.

### Agreement Between Left and Right Ankle Joint in Healthy Subjects

Radiographic parameters of the left and right ankle joint of the same healthy subject, including all 3 torques, showed excellent agreement for AD, PD, and ER with ICCs of 0.962 (95% CI 0.929-0.980; *P* < .01), 0.983 (95% CI 0.968-0.991; *P* < .01), and 0.983 (95% CI 0.967-0.991; *P* < .01), respectively. For the TCS, the ICC was moderate at 0.632 (95% CI 0.404-0.786; *P* = .37). For the β angle, the ICC was moderate at 0.73 (95% CI 0.551-0.849; *P* = .06).

## Discussion

The main finding of this study is that the contralateral ankle joint can be used as a reference for external torque CT assessment of syndesmotic stability as intraindividual side differences are negligible. Therefore, the hypothesis was confirmed.

Although large interindividual differences were found for AD (up to 3.2 mm), TCS (up to 4.7 mm), PD (up to 8.2 mm), ER (up to 12.4 degrees), and β angle (up to 16 degrees), which is in line with other studies,^3,6,7,9^ the intraindividual comparison of both ankle joints showed maximum differences of 0.5 mm for AD, 2.2 mm for TCS, 1 mm for PD, 3.1 degrees for ER, and 8.7 degrees for β angle.

Several studies have investigated different diagnostic methods to detect distal tibiofibular syndesmotic injuries. However, the diagnosis remains challenging because even subtle instabilities can be significant; the problem itself is multiplanar, and can often only be delineated with weightbearing.^4,12-14^ Diagnostic accuracy is further compromised by large interindividual variability, necessitating comparison with the contralateral side.^8,22,26^ Although a recent study^5^ showed reliable results for the uninjured syndesmosis using a combination of axial force and conventional CT scan to assess syndesmotic stability, several authors suggest the use of external rotational force rather than an axial force for assessment.^16,18,21^ The reason for this is that evaluation of the syndesmosis with axial loading alone using weightbearing plain radiographs or CT scans does not provide reliable results.^14,27,31^ Thus, the decision as to whether a syndesmotic injury is ultimately stable or unstable is made far too late, at the time of diagnostic arthroscopy, the current gold standard of diagnosis, and is left to the discretion of the treating surgeon.

Recognizing this dilemma, Beeler et al^1^ developed an external torque device that applies a predefined but variable external torque to the syndesmosis during supine CT. This new diagnostic tool was compared to diagnostic arthroscopy in a cadaveric study. Overall, the external torque device provided noninvasive, reliable detection of syndesmotic instability based on the “healthy” contralateral side. The sensitivity for detecting syndesmotic lesions with the external torque device was higher and the specificity comparable to arthroscopy. External torque showed a steady increase in fibular ER and posterolateral translation (PD, TCS), a trend that was logically not observed in the healthy subjects of the present study because the syndesmosis was stable. In agreement with the results of the latter study,^1^ the AD—combining rotation and translation of the fibula—seems to be the most reliable measure, with a maximum side difference of 0.5 mm in stable ankle joints. In the biomechanical cadaver study, syndesmotic injury was suspected with a sensitivity of 84.1% and a specificity of 95.2% when the side difference of AD was ≥1 mm. In this regard, it can be concluded that the healthy contralateral side is a valid reference when using the AD.

Among the radiologic parameters, the β angle and TCS showed the greatest intraindividual variability for all 3 applied torques, with a moderate agreement between the left and right ankle joint. The other 3 parameters showed excellent agreement between left and right, with ER showing larger side differences of up to 3 degrees compared with the other 2 parameters. This suggests that the combined measurement of AD and PD may be the safest combination for the detection of syndesmotic lesions in clinical application. Exact cutoff values cannot be derived from the results of the present study, but considering the 95% range of the data and all 3 torques, the examiner should consider a syndesmotic injury if the side difference is greater than 0.5 mm for AD and greater than 0.9 mm for PD.

### Limitations

The present study demonstrates the limitations of a biomechanical study. The selection of external torque levels was made arbitrarily, yet with consideration for what would be feasible in a clinical setting. Although external torques of up to 7.5 Nm could be applied without pain in healthy subjects, this may not be possible in patients with syndesmotic injuries and could also have a negative effect through increased muscular counteracting to reduce pain. Although AD, ER, and the β angle slightly increased with increasing external torque, TCS and PD decreased. However, the amount of external torque did not affect the differences to the contralateral side. This suggests that even the application of 2.5 Nm may be sufficient to detect subtle instabilities. However, this needs to be confirmed in patients with syndesmotic insufficiency. Furthermore, the possibility of intraarticular infiltration with local anesthetic to reduce pain remains. In addition, stabilization of the knee joints with a strap to reduce the external rotation of the hip joints could show some variability (varying tension of the strap). However, the final external torque applied to the ankle should be the same. The threshold for clinical relevance of syndesmotic instability remains unclear, but the observed intraindividual left-right differences may provide guidance for establishing such a threshold. Overall, further clinical studies with the external torque device in patients are essential.

## Conclusion

Using the healthy contralateral ankle joint is appropriate for assessing syndesmotic stability based on minimal intraindividual side differences using the external torque device. Side differences greater than 0.5 mm for AD and greater than 0.9 mm for PD may be considered abnormal. However, future studies are needed to define definitive cutoff values for relevant side differences in acute and chronic syndesmotic instability to guide clinicians in their treatment decisions.

## Supplemental Material

sj-pdf-1-fai-10.1177_10711007241262771 – Supplemental material for The Contralateral Ankle Joint Is a Reliable Reference for Testing Syndesmotic Stability Using Bilateral External Torque CT Supplemental material, sj-pdf-1-fai-10.1177_10711007241262771 for The Contralateral Ankle Joint Is a Reliable Reference for Testing Syndesmotic Stability Using Bilateral External Torque CT by Anna-Katharina Calek, Esteban Ongini, Bettina Hochreiter, Reto Sutter, Stephan H. Wirth and Silvan Beeler in Foot & Ankle International
